# Treatment of Recurrent Nasopharyngeal Carcinoma: A Sequential Challenge

**DOI:** 10.3390/cancers14174111

**Published:** 2022-08-25

**Authors:** Zhouying Peng, Yumin Wang, Ruohao Fan, Kelei Gao, Shumin Xie, Fengjun Wang, Junyi Zhang, Hua Zhang, Yuxiang He, Zhihai Xie, Weihong Jiang

**Affiliations:** 1 Department of Otolaryngology Head and Neck Surgery, Xiangya Hospital, Central South University, Changsha 410008, China; 2 Otolaryngology Major Disease Research Key Laboratory of Hunan Province, Changsha 410008, China; 3 National Clinical Research Center for Geriatric Disorders, Xiangya Hospital, Central South University, Changsha 410008, China; 4 Anatomy Laboratory of Division of Nose and Cranial Base, Clinical Anatomy Center of Xiangya Hospital, Central South University, Changsha 410008, China; 5 Department of Radiation Oncology, Xiangya Hospital, Central South University, Changsha 410008, China

**Keywords:** recurrent, nasopharyngeal carcinoma, treatment modality, management, biomarkers

## Abstract

**Simple Summary:**

Recurrent nasopharyngeal carcinoma is one of the major causes of death among NPC patients. However, there are no international guidelines for the treatment of patients with recurrent NPC now. In this article, we summarize past publications on clinical research and mechanistic studies related to recurrent NPC, combined with the experience and lessons learned by our institutional multidisciplinary team in the treatment of recurrent NPC. We propose an objective protocol for the treatment of recurrent NPC.

**Abstract:**

Recurrent nasopharyngeal carcinoma (NPC), which occurs in 10–20% of patients with primary NPC after the initial treatment modality of intensity-modulated radiation therapy (IMRT), is one of the major causes of death among NPC patients. Patients with recurrent disease without distant metastases still have a chance to be saved, but re-treatment often carries more serious toxicities or higher risks. For this group of patients, both otolaryngologists and oncologists are committed to developing more appropriate treatment regimens that can prolong patient survival and improve survival therapy. Currently, there are no international guidelines for the treatment of patients with recurrent NPC. In this article, we summarize past publications on clinical research and mechanistic studies related to recurrent NPC, combined with the experience and lessons learned by our institutional multidisciplinary team in the treatment of recurrent NPC. We propose an objective protocol for the treatment of recurrent NPC.

## 1. Introduction

Nasopharyngeal carcinoma (NPC) is a squamous carcinoma of the head and neck with great geographical distribution and ethnic heterogeneity, which affects populations predominantly in Southeast Asia, with the highest incidence in southern China [1,2,3,4]. Radiotherapy is recommended as the main treatment for primary NPC, and with the development of integrated treatment techniques such as radiotherapy combined with chemotherapy and targeted therapy, the 5-year overall survival (OS) rate for NPC can reach 50–64%, but 10–20% of patients still experience recurrence after the first treatment and improvement of their disease [5,6,7,8,9,10,11].

According to the National Comprehensive Cancer Network (NCCN) guidelines, for patients with resectable head and neck squamous carcinoma who have received radiotherapy, surgery to remove the lesion or local radiotherapy is recommended after recurrence, while chemotherapy alone is usually palliative care for those who are not suitable for radiotherapy or surgery [12,13]. Finding optimal treatment strategies for patients with recurrent NPC to prolong their survival after recurrence and improve their survival and quality of life has been the concern of otolaryngologists and oncologists in recent years [14,15].

There are many high-quality publications that have compared the efficacy of various treatment options [16,17,18,19,20]. In this review, we have discussed the current issues and summarized the cutting-edge views on the diagnosis and treatment of recurrent NPC.

## 2. Clinical Symptom and Diagnosis of Recurrent NPC

**Figure 1:**
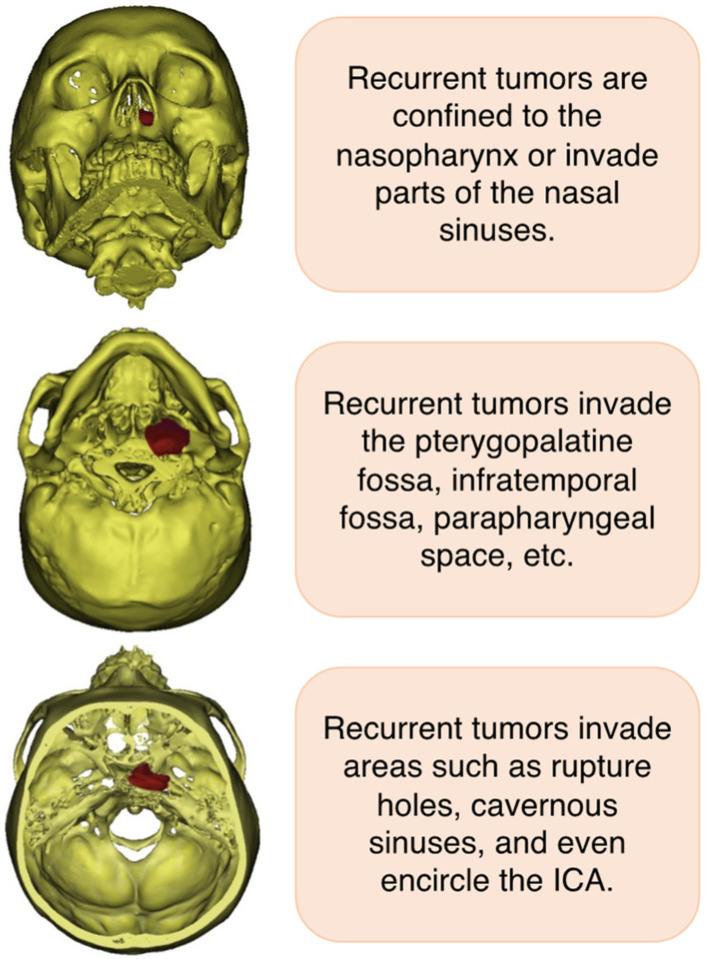
The common sites of tumor involvement in recurrent NPC. NPC, nasopharyngeal carcinoma; ICA, internal carotid artery.

The common clinical manifestation of recurrent NPC is blood accumulation in the nose or sputum, which is different from primary NPC, where the most common clinical symptom is neck swelling, while ear symptoms and headache are also more common [1,21]. In the case of recurrent NPC, it is not uncommon to have corresponding dysfunction of cranial nerves, muscles or adjacent organs of the nasopharynx after they have been invaded by the tumor [2,22]. Patients with cervical lymph node metastasis at the time of the first diagnosis are more likely to have a recurrence with a neck mass [6,9]. The 8th edition of the Union for International Cancer Control (UICC) and the American Joint Committee on Cancer (AJCC) TNM staging criteria for NPC are used as the latest standards for diagnosis in the clinic [23,24]. In contrast, the 6th or 7th edition of the TNM staging criteria for NPC is more commonly used by researchers in previous publications [6,7,9,25,26,27]. The 6th or 7th edition staging may be more applicable to recurrent NPC. According to these editions, NPC is characterized using the following stages: 1. rT1—tumor is confined to the nasopharynx; 2. rT2—tumor has invaded the nasal cavity, sinuses, parapharyngeal space, and/or adjacent soft tissues (internal pterygoid muscle, medial pterygoid muscle, etc.); 3. rT3—tumor has invaded the base wall of the pterygoid sinus; 4. rT4 tumor has invaded the intracranial structures, internal carotid artery, cranial nerves, orbit, parotid gland, external pterygoid muscle, and other adjacent tissues in a larger area. Figure 1 shows the common sites of tumor involvement in recurrent NPC. The gold standard for the diagnosis of recurrent NPC is pathological diagnosis [28,29]. Compared with primary NPC, the difference is that the main body of the lesion is not in the nasopharynx or nasal cavity, which are convenient for biopsy, but in the parapharyngeal space or adjacent muscular tissues or even in the intracranial area, where the traditional transnasal biopsy is not so applicable. Therefore, in the diagnosis of recurrent NPC, imaging has a higher impact. The most commonly used imaging techniques to evaluate the lesion before treatment are: high-resolution computed tomography (HRCT) of the nasopharynx and skull base, enhanced magnetic resonance imaging (MRI) of the nasopharynx and skull base, and positron emission tomography CT (PET-CT) of the whole body.

### 2.1. High-Resolution CT

**Figure 2:**
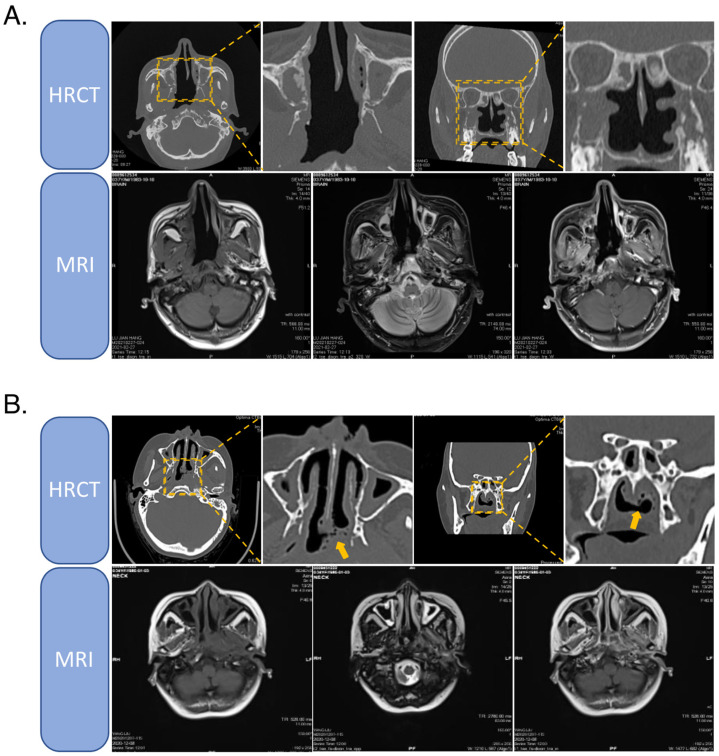
(**A**) HRCT and MRI images of bone necrosis in recurrent NPC patients. (**B**) HRCT and MRI images of soft tissue necrosis in recurrent NPC patients. An “air bubble shadow” can be seen in the soft tissue shadow on CT.

High-resolution CT examination has its advantages for the diagnosis of nasopharyngeal skull base disease, especially the malignant lesions, to determine the degree and extent of bone destruction. Compared with conventional plain scan and/or CT enhancement, high-resolution CT is more advantageous in discriminating bone destruction [30,31]. During the diagnosis of recurrent NPC, it is more helpful to identify whether it is tumor recurrence or bone and/or soft tissue necrosis after radiotherapy. If the carcinoma has recurred, the bone on CT will be predominantly eroded by the tumor tissue unless the lesion is extensive, and generally, the CT changes will be concentrated on a single bone. In contrast, if there is only post-radiation bone and/or soft tissue necrosis, it will appear mainly as “spongy” changes of extensive bone centered on the pterygoid bone on high-resolution CT. However, there is less evidence on tumor erosion and destruction of the bone, especially of the bone cortex to the point of discontinuity [32]. In cases of severe soft tissue necrosis, an “air bubble shadow” (Figure 2) is more often seen in the soft tissue shadow on CT scans [32].

### 2.2. Enhanced MRI

Enhanced MRI of the nasopharynx and skull base is essential for the diagnosis and differential diagnosis of recurrent NPC. Similarly to most malignant tumors, NPC has a high signal in enhanced MRI and generally a low signal in T1- and T2-weighted levels [33]. In addition to assisting in determining the nature of the lesion, another important role of enhanced MRI is to help clinicians predict the extent of the tumor invasion and observe the relationship between the tumor and surrounding important structures [34]. It is important to note that patients with a suspected possibility of tumor recurrence have generally received radiation therapy, and sometimes there is a post-radiation effect that causes non-tumor tissues to appear as high-signal images in enhanced MRI, which may affect the clinician’s diagnosis [35]. Enhanced MRI also plays an important role in the post-treatment review process [36]. Whether the patient receives radiotherapy again after recurrence or undergoes nasopharyngeal surgery, enhanced MRI of the nasopharynx or even the skull base should be reviewed regularly after treatment to monitor the disease progression.

### 2.3. PET-CT

With its advent and development over the last decade, PET-CT has a unique advantage in the diagnosis of malignant tumors, especially in monitoring local progression and distant metastases [37,38,39,40]. The PET-CT approach involves the use of radionuclide labeling of compounds involved in human tissue metabolism, signal detection, and then image reconstruction [41]. This is used to distinguish tumor tissue with relatively high metabolism from non-tumor tissues with low metabolism [42,43]. However, its limitation is that some inflammatory areas will also show high metabolic signals, which are more likely to interfere with the judgment [44]. Therefore, at present, PET-CT is still more often used to exclude distant metastatic foci.

For recurrent NPC, there are other methods for diagnosis and prognosis in addition to the diagnostic and differential roles of conventional imaging approaches described above. In recent years, some researchers have also explored radiomics-related differential diagnostic methods or means of predicting prognosis [36,45,46,47,48]. There have been many reports on the use of imaging histology in the diagnosis of head and neck tumors as well as imaging histology combined with molecular studies to explore the prognosis of malignant tumors. Peng et al. summarized the application of imaging histology in head and neck malignancies in the past 10 years [3]. The use of imaging histology in recurrent NPC has been less frequently published. Zhang et al. constructed a predictive model for recurrence-free survival after first treatment in patients with stage T4 primary NPC by extracting features from tumor regions in MRI findings for 360 patients with primary NPC and validating them with a training cohort [48]. Furthermore, a machine learning approach based on PET-CT was used to identify recurrence and inflammatory changes after NPC treatment [49].

## 3. Treatment of Recurrent NPC

For recurrent NPC, the main treatment options currently include surgical resection and re-irradiation with or without chemotherapy, while chemotherapy alone is generally recommended only for patients who are unable to undergo surgical resection or who cannot tolerate secondary radiation therapy [8,13,50,51]. For resectable recurrent NPC, the 2- and 5-year OS rates for salvage nasopharyngectomy ranged from 48.6% to 100.0% and 38.3% to 88.9%, respectively [17,52,53,54]. If re-radiation was performed, the 2- and 5-year survival rates were 44.3–77.7% and 27.5–57.2%, respectively [55,56]. It is clear that the prognosis in recurrent patients is poor compared to that in primary cases, regardless of the treatment modality applied [1]. In the last decade, with the development of multimodal treatments with different combinations of chemotherapeutic agents, targeted therapy, and immunotherapy, other viable treatment options are being provided for patients with locally advanced recurrent malignancies [57,58,59,60]. However, there are still no clear multimodal treatment options for patients with recurrent NPC due to the lack of strong evidence-based medical findings. By synthesizing the currently available research publications, we have summarized and outlined the main and newer therapeutic approaches currently available for recurrent NPC.

### 3.1. Re-Irradiation

Re-irradiation is a very important treatment for recurrent NPC, but it is also accompanied by difficulties and challenges [22]. The difficulty lies in the fact that the re-irradiation dose is difficult to control due to the toxic side effects after the initial treatment, and the dose must be tuned to achieve the effective dose value while minimizing the toxic effects after radiotherapy [22]. The scope and location of recurrent tumors are more “individual” than the primary tumors, with more adjacent important tissues and more difficulty involved in judging the imaging [2]. For radiologic oncologists, a balance must be found between prolonging the survival in patients with recurrent NPC and the development of severe post-radiotherapy toxic effects. With the advent and development of IMRT, Qiu et al. compared the efficacy of 3D conformal radiation therapy with IMRT for the treatment of locally recurrent NPC [61]. They found that IMRT had similar efficacy and lower incidence of toxic side effects than 3D conformal radiation therapy did.

In an international recommendation for the treatment of recurrent NPC using IMRT published in 2020, oncologists from around the world agreed that IMRT should be the preferred modality for radiotherapy of recurrent NPC, but have not yet completely rejected the availability of brachytherapy and stereotactic radiotherapy for small recurrent foci. This publication compiled some of the aspects of contention regarding IMRT for recurrent NPC, and a questionnaire was also distributed to more than 20 internationally recognized experts for their opinions. Then, the results were collected and weighed to draw relevant conclusions about the diagnosis and treatment. The consensus discussed the delineation of clinical target volume (CTV) and planning target volume (PTV), with the preferred CTV to be added to the gross tumor volume (GTV) with a margin of less than approximately 5 mm. In terms of the total dose of re-radiotherapy, the most commonly used total dose is ≥60 Gy, and the efficacy will be reduced if the total dose is less than 60 Gy. If the total dose of re-radiotherapy exceeds 68 Gy, there is no significant increase in OS rates, and it is more likely to have fatal complications [22]. This paper summarizes the use of IMRT for recurrent NPC in the last 10 years since its widespread use (Table 1). In recent years, with the development of various forms of novel radiotherapy, there have also been reports related to carbon ion treatment for recurrent NPC [62,63,64,65]. However, this type of treatment is not yet mainstream, and longer-term observations of efficacy will be needed to determine whether patients can benefit from this type of treatment in total.

### 3.2. Surgical Resection

With the development of surgical techniques and the increased understanding of the anatomy of the eustachian tube region by otolaryngologists, surgical resection of the tumor has become another important option for the treatment of recurrent NPC [77,78,79]. Surgical approaches include open surgical approaches such as transoral and maxillary external rotation and endoscopic transnasal approaches [80]. Li et al. conducted a meta-analysis of endoscopic versus open surgical treatment of recurrent NPC and showed that in patients with rT3, the 2-year OS rate was 67% versus 53% for endoscopic versus open surgical treatment, respectively [52]. Compared to open surgery, nasal endoscopic resection of nasopharyngeal lesions has the advantages of lower trauma, faster recovery, no postoperative facial scar formation, better functional protection, and lower cost. There is a lack of additional evidence to support that endoscopic nasopharyngeal tumor resection is necessarily better than open surgery. However, with the development of endoscopic techniques and surgical instruments, open surgery to remove nasopharyngeal lesions is gradually being replaced. For surgeons, it is also important to focus on how to develop indications for endoscopic surgery for patients with recurrent NPC. The criteria commonly used in current publications to consider the difficulty of endoscopic surgery are mainly rT stage and tumor invasion of the surrounding vital structures, with rT stage being shown in several studies to be an independent factor that can affect patient survival [7,9]. Most of the available studies have focused on nasal endoscopic surgery for recurrent patients with rT1–rT3 [6,16,20,78,79,81]. Liu et al. published a report on a total of 96 patients treated with rT1–rT3 nasal endoscopic surgery with 2- and 5-year OS rates of 89.9% and 73.8%, respectively [6]. Whether the surgery has a negative margin, or whether patients with a positive margin receive postoperative treatment, also has a significant impact on the patient’s prognosis [9,82]. In patients with rT4, which is a major challenge for both oncologic radiologists and ENT surgeons, there are not many summaries of nasal endoscopic resection of recurrent nasopharyngeal carcinoma among the available publications, and the efficacy of surgery is significantly lower than that for patients with rT1–rT3. Publications by Wong et al. and Peng et al. reported 2-year OS rates of 66.7% and 35.6% for rT3–rT4 patients, respectively, showing that the survival rate for rT3–rT4 patients was significantly worse than that for rT1–rT2 patients [7,25]. We have summarized the publications on nasal endoscopic nasopharyngeal lesion resection for recurrent NPC reported in the last 10 years (Table 2).

It is evident that in patients with recurrent NPC in rT3–rT4, not only is the prognosis relatively poor, but the treatment challenges are greater, regardless of whether they are treated with re-radiotherapy or surgical resection. How to better prolong the survival in these patients requires surgeons who are familiar with the anatomy of the nasopharynx and have extensive surgical experience. Preoperative evaluation and postoperative monitoring are also very important. Especially in patients with recurrent tumors closely related to the internal carotid artery (ICA), preoperative vascular assessment and preoperative vascular pretreatment are extremely important to completely remove the tumor and to avoid intraoperative and postoperative fatal complications. At our institution, carotid endothelial imaging will be performed in these patients to determine the extent of tumor invasion more carefully. In patients with ICA invasion, a digital subtraction angiography (DSA) and balloon occlusion test (BOT) will be performed to evaluate the compensatory status of the healthy vessels in the segment with blockage of the affected ICA [91,92]. The affected ICA (already occluded) can then be removed along with the tumor [93]. If the BOT is positive, a neurosurgeon may be called in to assist with cerebral artery bypass surgery to replace blood flow to the affected ICA [94,95].

### 3.3. Chemotherapy, Targeted Therapy, and Immunotherapy

Chemotherapy is one of the most classical treatment modalities for malignant tumors, and depending on the type of tumor, chemotherapy can play a “leading” or “supporting” role in the treatment process [96,97,98,99,100]. For primary NPC, radiotherapy with chemotherapy is almost the accepted treatment modality, but the “role” of chemotherapy in the treatment of recurrent NPC is still debatable [101,102,103]. Currently, several publications have reported on the treatment of recurrent NPC using different chemotherapy regimens; many oncologists believe that for patients with rT1–rT2, retreatment with radiotherapy or surgery is an option. However, for patients with rT3–rT4 tumors, the radiation dose of radiotherapy alone is often not ideal for tumor volume coverage, and the 5-year survival rate is less than 35% [15,22]. In addition, when the tumor is close to vital organs and structures, the increase in radiation dose may trigger serious or even lethal complications after radiotherapy [104,105]. In order to allow the use of relatively safe and effective re-radiotherapy, some studies have shown that cisplatin, docetaxel, and fluorouracil (TPF) induction therapy is a wise choice [106,107]. However, it has also been suggested that poor compliance with cisplatin in some patients treated with TPF may necessitate new chemotherapy regimens to replace TPF chemotherapy regimens [108,109]. Nedaplatin (S-1) is considered a safe and effective treatment option for patients with recurrent NPC after failure of platinum-based chemotherapy [110,111]. The clinical trials of Chen et al. and Hong et al. showed the same results, and gemcitabine plus cisplatin (GP) chemotherapy regimens are increasingly considered as the first-line chemotherapy regimens for the treatment of recurrent NPC [112,113,114]. As for oral chemotherapy drugs, they are generally used when patients cannot tolerate intravenous drugs, and can also be used as a maintenance treatment option for patients with tumor.

Compared with chemotherapeutic agents, the use of targeted therapy and immunotherapy in recurrent NPC is relatively limited, and in most cases they are not used alone, but in combination with chemotherapeutic agents or radiotherapy to provide better benefits [115,116,117,118,119]. A clinical study published by Ng et al. showed that weekly IMRT with docetaxel plus cetuximab was shown to be effective in advanced recurrent NPC compared to TPF-induced chemotherapy, with a 3-year progression-free survival rate of 35.7% [107]. The results of a multicenter, double-blind phase III clinical trial published in Lancet Oncology in 2021 showed that the GP chemotherapy regimen plus camrelizumab had acceptable toxicity and was effective in prolonging survival in patients with recurrent or metastatic NPC, making it a promising first-line treatment option for patients with recurrent NPC [120]. Several ongoing clinical trials are available on the Clinical trials website (https://clinicaltrials.gov accessed on 22 June 2022), all of which are concerned with immunotherapy with or without IMRT.

The efficacy of chemotherapy in combination with immunotherapy plus endoscopic surgery for recurrent NPC has not been reported in the literature. A recent single-arm phase II clinical trial (NCT05011227, NCT04778956) was hosted by Fudan University Eye, Ear, Nose and Throat Hospital and First Affiliated Hospital, Sun Yat-Sen University. They are recruiting patients with stage rT2 or higher resectable recurrent NPC with preoperative and postoperative immunotherapy alone or immunotherapy in combination with chemotherapy.

**Figure 3:**
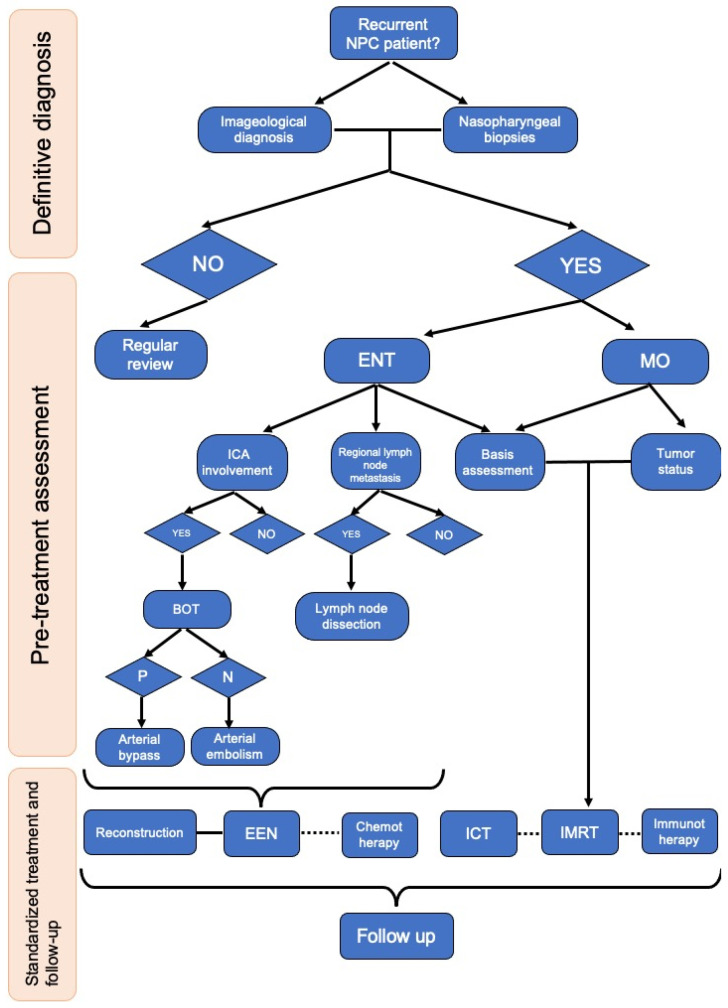
Flowchart for the diagnosis and treatment of recurrent NPC with post-treatment management. ENT, ear nose and throat; MO, medical oncology; ICA, internal carotid artery; BOT, balloon occlusion test; P, positive; N, negative; EEN, endoscopic endonasal nasopharyngectomy; ICT, induction chemotherapy; IMRT, intensity-modulated radiation therapy.

Regardless of the treatment modality, it is inevitable that patients with recurrent nasopharyngeal cancer will experience adverse symptoms after treatment, but oncologists and otolaryngologists should minimize the occurrence of fatal complications such as nasopharyngeal hemorrhage. The summary of the complications after treatment of recurrent NPC in the past 10 years (Table 3) made it clear that certain complications such as nasopharyngeal necrosis, osteonecrosis, and bleeding cannot be avoided regardless of IMRT or endoscopic surgical treatment. Therefore, for patients with recurrent NPC, applying the principle of early prevention and early detection and treatment, as well as follow-up after treatment may have the effect of prolonging the survival period and improving the quality of survival. By summarizing our institution’s treatment experience and that found in the published studies, we developed a flowchart for the diagnosis and treatment of recurrent NPC with post-treatment management (Figure 3).

### 3.4. Biomarkers of Recurrent NPC

**Figure 4:**
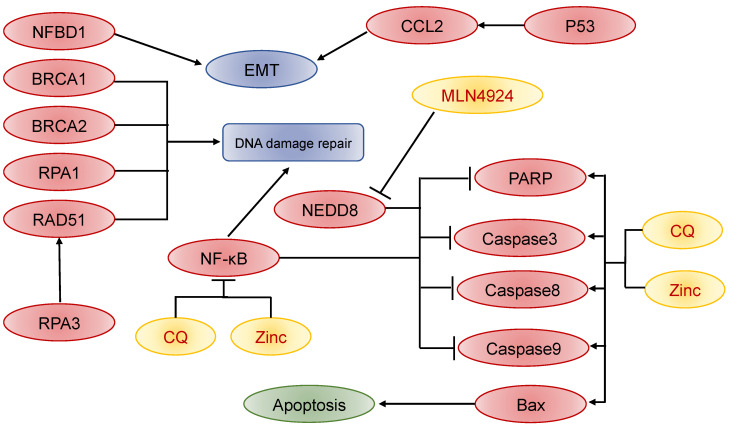
Related molecules and pathways affecting NPC recurrence and progression, such as invasive metastasis and radio resistance.

Radiotherapy plus chemotherapy is the first choice of treatment for recurrent NPC, and its effect is worthy of recognition, but tumor recurrence after re-radiotherapy is indeed the main reason for treatment failure. Guo et al. found that high expression of CCL2 was an independent prognostic factor for the survival of recurrent NPC without distant metastases, and that increased autocrine CCL2 secretion by tumors was positively correlated with protein phosphorylation, thus possibly enhancing the resistance of tumor cells to radiotherapy [121]. While ionizing radiation can induce tumor DNA damage, some small molecule drugs were experimentally shown to reduce the enrichment of NFBD1, RAD51, and BRCA1 at DSB repair sites by inhibiting NF-κB, thus slowing down DNA damage repair in tumor cells and improving radiotherapy sensitivity [122]. Autophagy of tumor cells is one of the most fundamental pathways leading to apoptosis, and it has been shown that NEDD8 can promote apoptosis through activation of caspase-3, caspase-8, caspase-9, and PARP, thereby increasing the sensitivity of tumor cells to radiotherapy and chemotherapy [123]. Liu et al. found that leukemia inhibitory factor (LIF) in the cytoplasm of NPC tumor cells and its receptor LIFR were associated with NPC tumor recurrence and poor prognosis, and further mechanistic studies showed that it affected NPC recurrence and invasive metastasis through SRC/PXN/FAK, TKS5/CTTN/MMP2 and VIM/N-cad (Figure 4) [124,125,126,127]. However, the mechanism of recurrent NPC occurrence and development has not been studied in depth, and more unknown areas need to be explored by medical doctors.

## 4. Discussion

As a refractory disease, recurrent NPC requires multidisciplinary team involvement from diagnosis to treatment and individualized treatment plans based on the patient’s condition. Single types of imaging have difficulty in identifying recurrence and soft tissue necrosis in many cases, although the gold standard of pathological biopsy still exists. However, there are often cases of recurrence where it is difficult to clip a biopsy. This can indeed pose a considerable challenge to physicians when developing a treatment plan.

When developing treatment plans for recurrent NPC, it is necessary to consider the patient’s own tolerance of treatment to maximize the patient’s benefit. Especially in the case of advanced rT3–rT4 patients, there is a risk of ICA damage and ICA-related bleeding during treatment. This often requires a team of nasocranial surgeons, oncologists, interventionalists, and neurosurgeons to develop a treatment plan. It is also important to follow up patients with recurrent NPC after they have received re-treatment. Our institution requires that all patients who undergo surgical treatment in our department have reviews with enhanced MRI of the nose and skull base every 3 months for 1 year, or every 6 months for 3 years, and maintain the frequency of review every 1 year for 5 years and seek medical consultation promptly when new symptomatic changes occur. Since patients with recurrent NPC have undergone at least one radiation treatment, and some have even undergone re-radiotherapy, the moisturizing and drainage functions of the nasal mucosa are lost; therefore, long-term nasal irrigation and the use of nasal oil drops can be of great help to patients in reducing symptoms such as nasal dryness and headache.

## 5. Conclusions

The diagnosis, treatment, and post-treatment management of recurrent NPC pose a current challenge for oncologists and otolaryngologists. Thus, clinicians need to weigh the benefits and drawbacks of various treatment approaches for patients. For the treatment of recurrent NPC, the best approach is to develop a relatively standard and industry-approved treatment plan through a joint evaluation of the patient by the oncologist and otolaryngologist. On the basis of the standard treatment process, each patient will be treated according to his or her own personalized plan after optimization of the unified plan, in order to prolong the patient’s survival and improve his or her quality of life.

## Figures and Tables

**Table 1:** Characteristics of the literature on IMRT for recurrent NPC in the last 10 years.

	Year	No. of Patients	M/F	rT Classification		ReRT Mean Dose (Gy)	CCT	OS Rate (%)			DFS (%)		LCR/LRFS (%)		Median Follow-Up Time (Months)
				rT1-2	rT3-4			2-Year	3-Year	5-Year	2-Year	5-Year	3-Year	5-Year	
Qiu et al. [29]	2012	70	56/14	30	40	70.0 (50.0–77.4)	18	67.4	-	-	65.8	-	-	-	25.0 (3.0–80.0)
Han et al. [66]	2012	239	182/57	59	180	70.0 (61.7–77.5)	117	-	-	44.9	-	45.4	-	85.8	12.0 (3.0–62.0)
Hua et al. [67]	2012	151	122/29	29	122	70.4 (62.1–77.6)	37	-	46.4	38.0	-	-	83.2	80.7	40.0 (7.2–116.9)
Chen et al. [68]	2013	54	44/10	11	43	70.0 (49.8–76.6)	18	44.3	-	-	-	-	-	-	16.5 (1.0–93.0)
Tian et al. [69]	2013	251	195/56	53	198	70.7 (61.1–79.7)	126	-	53.2	41.1	-	-	80.6	75.1	40.0 (3.0–147.0)
Karam et al. [70]	2015	27	20/7	21	6	54.0 (39.0–97.0)	23	-	53.0	-	-	-	53.0	-	36.0
You et al. [16]	2015	72	18/54	59	13	-	42	-	-	55.5	50.0	-	-	82.3	49.4 (3.1–149.0)
Zou et al. [20]	2015	218	173/45	57	161	-	84	-	-	39.0	-	-	-	-	33.0 (2.0–146.0)
Xiao et al. [71]	2015	291	225/66	47	244	-	120	-	-	33.2	-	-	-	66.6	29.0 (3.1–146.0)
Chan et al. [72]	2016	38	31/7	0	38	-	8	-	47.2	-	-	-	44.3	-	47.8 (13.5–118.1)
Tian et al. [73]	2017	245	196/49	0	245	70.0 (60.1–78.7)	157	-	-	27.5	-	-	-	60.9	24.0 (2.0–132.0)
Ng et al. [74]	2017	33	-	0	33	-	-	-	63.8	-	-	-	49.2	-	28.5
Kong et al. [75]	2018	184	133/51	64	120	66.7 (42.0–77)	138	-	46.0	28.8	-	-	85.1	71.1	32.0 (3.0–125.0)
Zhang et al. [76]	2019	44	33/11	21	23	66.0 (54.0–70.0)	33	-	56.8	-	-	-	58.9	-	28.0 (5.0–168.0)
Liu et al. [6]	2021	100	72/28	69	31	-	-	77.7	68.0	57.2	81.8	59.0	89.8	77.0	-

IMRT, intensity-modulated radiotherapy; NPC, nasopharyngeal carcinoma; M, male; F, female; ReRT, re-radiotherapy; CCT, concurrent chemotherapy; OS, overall survival; DFS, disease-free survival; LCR, local control rate; LRFS, local recurrence-free survival.

**Table 2:** Characteristics of the literature on EEN for recurrent NPC in the last 10 years.

Authors	Year	NO. of Patients	M/F	rT Classifications		Margin(+/−)	Margins+ Therapy	OS Rate (%)		DFS Rate (%)		Mean Follow-Up Time (Months)
				rT1-2	rT3-4			2-Year	5-Year	2-Year	5-Year	
Ho et al. [83]	2012	13	9/4	11	2	4/9	Y	100	-		-	24.2
Castelnuovo et al. [84]	2013	27	-	13	14	3/24	-	81.5	75.1	70.4	58.1	31.2
Emanuelli et al. [85]	2014	8	6/2	8	0	-	-	100	-	88.9	-	27.0
You et al. [16]	2015	72	54/18	59	13	-	-	-	77.1	-	-	-
Zou et al. [20]	2015	92	22/70	79	13	-	-	-	78.1	-	-	-
Chen et al. [86]	2015	96	72/24	38	58	51/44	-	51.7	-	-	-	-
Wong et al. [27]	2016	15	-	0	15	6/9	Y	66.7	-	40.0	-	28.7
Vlantis et al. [87]	2016	18	11/7	18	0	2/16	N	100	88.9	90.0	-	22.0
Weng et al. [19]	2017	36	26/10	17	19	-	-	66.5	-	64.0	-	54.0
Liu et al. [88]	2017	91	71/20	43	48	-	-	64.8	38.3	57.5	30.2	median 23.0
Sun et al. [89]	2015	71	53/18	37	34	17/20	-	56.3	-	-	-	-
Tang et al. [82]	2019	55	44/11	45	10	4/51	-	98.2	-	-	-	18.0
Wong et al. [25]	2019	12	-	0	12	-	-	-	50	-	25	44.8
Li et al. [9]	2020	189	132/57	97	92	32/157	-	82.2	43.6	-	-	median 24.0
Thamboo et al. [90]	2021	13	9/4	11	2	3/10	Y	100	84.6	76.9	53.8	74.3
Liu et al. [6]	2021	96	-	66	30	6/90	-	89.9	73.8	81.8	59.0	median 56.0
Peng et al. [7]	2021	56	38/18	13	43	-	-	48.6	-	42.6	-	44.0

EEN, endoscopic endonasal nasopharyngectomy; NPC, nasopharyngeal carcinoma; M, male; F, female; Y, yes; N, no; OS, overall survival; DFS, disease-free survival.

**Table 3:** Complications of the two main treatment modalities for recurrent NPC.

Complications	No. of Patients in IMRT (Median, Range, %)	No. of Patients in EEN (Median, Range, %)
Cranial nerve palsies	11.4 (7.1–28.6)	13.9
Headache	17.3 (8.7–23.1)	24.0 (9.7–25.0)
Trismus	22.2 (7.9–46.2)	9.7
Deafness	29.3 (4.5–65.4)	11.1 (6.9–34.0)
Otitis media	23.1	30.8 (25.0–70.0)
Necrosis (including ON, TLN, NN)	46.2 (3.6–63.7)	23.1 (6.9–44.4)
Dysphagia	17.3 (5.5–26.3)	8.3 (7.7–9.3)
Hemorrhage	15.8 (11.5–17.2)	6.0 (2.8–9.9)
Sinusitis	40.6	12.0 (5.0–15.4)
Xerostomia	30.8	11.1–17.9
Neck fibrosis	26.3 (0.5–34.6)	-
Cachexia	15.3	4.2–10.7

NPC, nasopharyngeal carcinoma; ON, osteoradionecrosis; TLN, temporal lobe necrosis; NN, necrosis of nasopharynx.

## Data Availability

Not applicable.
